# Efficacy and safety of fixed dose combination of Sitagliptin, metformin, and pioglitazone in type 2 Diabetes (IMPACT study): a randomized controlled trial

**DOI:** 10.1186/s40842-023-00161-6

**Published:** 2024-02-10

**Authors:** Mondal Aashish, Naskar Arindam, Sheelu Shafiq Siddiqi, Deepak Bhosle, V. J. Mallikarjuna, Dange Amol, Sorate Sanket, Gavali Omkar, Patel Parth, Hasnani Dhruvi, Prasad Durga, Dalwadi Pradeep, Kumar Suresh, Pathak Vaishali, Chaudhari Mayura, Basu Indraneel, Shembalkar Jayashri, Fariooqui Arif, S. K. Raghavendra, Varade Deepak, Thakkar Ravindra, Bhanushali Shaishav, Gaikwad Vijay, Kamran Khan, V. V. Mahajani, A. D. Sharma, Mayur Mayabhate, R. R. Pawar, A. S. Aiwale, Shahavi Vinayaka

**Affiliations:** 1 Medical College Kolkata, Kolkata, West Bengal India; 2 School of Tropical Medicine, Kolkata, West Bengal 700073 India; 3 Rajiv Gandhi Centre for Diabetes and Endocrinology, Aligarh Muslim University, Aligarh, Uttar Pradesh India; 4 Devgiri Diabetes Centre, Aurangabad, Maharashtra India; 5 Sadhana Speciality Clinic, Davanagere, Karnataka India; 6 Lifepoint Multispeciality Hospital, Wakad, Maharashtra India; 7 Sanjeevani Hospital, Nashik, India; 8 Lokamanya Multispeciality Hospital, Nashik, India; 9 Shushrusha Navneet Memorial Hospital, Ahmedabad, India; 10 Rudraksha Institute of medical Sciences, Ahmebabad, India; 11 Help Hospitals Pvt Ltd., Vijayawada, India; 12 Nirmal Hospital Pvt Ltd., Surat, India; 13 Panimalar Medical College Hospital and Research Institute, Chennai, Tamilnadu India; 14 Deoyani Multispeciality Hospital, Pune, India; 15 Ishwar Institute of Health Care, Aurangabad, Maharastra India; 16 Shubham Subhawana Superspeciality Hospital, Varanasi, India; 17 Getwell Hospital, Nagpur, India; 18 Landmark hospital, Hyderabad, India; 19 RHTC, Bangalore, India; 20 BAJRR Hospital and Research Centre, Dombivli, Maharashtra India; 21 VS general hospital, Ahmedabad, 380015 India; 22 AIMS hospital, Dombivli, India; 23 Govt Medical College, Jalgaon, India; 24 Nidan Kutir Diabetes, Bhagalpur, Bihar India; 25 Samarth Hospital Pvt Ltd., Satara, India; 26 Medical Affairs Department, Alkem Laboratories Ltd, Mumbai, 400013 India

**Keywords:** Type 2 diabetes mellitus, IMPACT study, Pioglitazone, Metformin, Sitagliptin, Triple therapy

## Abstract

**Background:**

Due to the progressive decline in β-cell function, it is often necessary to utilize multiple agents with complementary mechanisms of action to address various facets and achieve glycemic control. Thus, this study aimed to evaluate the efficacy and safety of a fixed-dose combination (FDC) of metformin/sitagliptin/pioglitazone (MSP) therapy vs. metformin/sitagliptin (MS) in type 2 diabetes mellitus (T2DM).

**Methods:**

In this phase 3, multicenter, double-blind study, patients with T2DM who exhibited inadequate glycemic control with HbA1c of 8.0–11.0% while taking ≥1500 mg/day metformin for at least 6 weeks were randomized to receive either FDC of MSP (1000/100/15 mg) or MS (1000/100 mg) per day for 24 weeks. The primary outcome measure was the change in HbA1c, and secondary outcomes included changes in fasting plasma glucose (FPG), postprandial plasma glucose (PPG), and body weight from baseline to 24 weeks along with safety and tolerability.

**Results:**

Among the 236 patients randomized, 207 (87.71%) successfully completed the study. All baseline characteristics were comparable between the FDC of MSP and MS groups. There was a subsequent significant reduction of HbA1c in FDC of MSP (− 1.64) vs. MS (− 1.32); between groups was [− 0.32% (95% CI, − 0.59, − 0.05)], *P* = 0.0208. Similar reductions were found in FPG [− 13.2 mg/dL (95% CI, − 22.86, − 3.71)], *P* = 0.0068, and PPG [− 20.83 mg/dL (95% CI, − 34.11, − 7.55)], *P* = 0.0023. There were no significant changes in body weight. A total of 27 adverse effects (AEs) and one severe AE were reported, none of which were related to the study drug.

**Conclusion:**

The FDC of MSP demonstrated significant efficacy in managing glycemic indices and could serve as a valuable tool for physicians in the management of Indian patients with T2DM.

**Trial registration:**

Clinical Trials Registry of India, CTRI/2021/10/037461.

## Background

Globally, the prevalence of type 2 diabetes mellitus (T2DM) is on the rise [1]. According to The International Diabetes Federation (IDF), it is projected that 783 million people will be diagnosed with T2DM globally by 2045 [2]. This progressive disease is characterized by multiple pathophysiologic abnormalities, including muscle insulin resistance, hepatic insulin resistance, adipocyte insulin resistance, progressive β-cell failure, apoptosis, increased α-cell secretion of glucagon, increased hepatic sensitivity to glucagon, reduced incretin effect due to β-cell resistance to glucagon-like peptide-1 (GLP-1) and gastric inhibitory polypeptide (GIP), increased renal glucose production, elevated renal tubular glucose reabsorption, brain insulin resistance, and altered neurotransmitter dysfunction, leading to impaired appetite suppression and weight gain, which are collectively referred to as ‘Ominous octet’ [3]. Recently, it was reported that insulin resistance in muscle and liver, along with β-cell failure, are the core pathophysiologic defects in T2DM. Several antidiabetic agents have been developed to target these defects, leading to improved glucose control in T2DM [3, 4].

Metformin is commonly used as a first-line therapy, but over time, it often fails to maintain adequate glycemic levels. It has been observed that treatment with a single antihyperglycemic agent is often unsuccessful in achieving and/or maintaining long-term glycemic control in patients with T2DM, leading to the need for combination therapies [5]. Different classes of drugs include thiazolidinedione, dipeptidyl peptidase-4 (DPP-4) inhibitor, sodium-glucose linked transporter-2 inhibitor, GLP-1 receptor agonist, and basal insulin, target different pathways to address the multiple pathophysiology of T2DM. These are recommended in combination with metformin to improve efficacy [1, 6].

Metformin prevents hepatic gluconeogenesis and glycogenolysis, increases liver and peripheral tissue sensitivity to glucose, and lowers Hb1Ac levels [7, 8]. Sitagliptin, a DPP-4 inhibitor, can raise blood levels of biologically active incretins, stimulating the release of insulin and attenuating the release of glucagon, primarily in response to a meal, which reduces glucose production in a glucose-dependent manner [9, 10]. One of the thiazolidinediones, pioglitazone, is a peroxisome proliferator-activated receptor γ (PPAR-γ) agonist that increases insulin sensitivity by improving insulin-mediated glucose elimination, leading to decreased plasma insulin concentrations [11]. It has also been demonstrated to improve β-cell responsiveness and increase β-cell function, suggesting that it may have an essential impact on reducing hepatic glucose production [12, 13]. Thus, pioglitazone is commonly used as an add-on medication when metformin, DPP-4 inhibitors, GLP-1 analogs, or their combination do not achieve the desired glycemic target [14].

Both pioglitazone and sitagliptin efficacy and safety have been well documented, proving that similar antidiabetic effects with distinct mechanisms of action may help to target various facets of ominous octet [8, 15]. Furthermore, the addition of pioglitazone alongside metformin and sitagliptin in triple oral therapy has been effective in glycemic control, addressing insulin resistance and islet β-cell dysfunction, which are the core defects in T2DM [7, 9, 15–17]. The advantage of combination therapy is that it helps to minimize the adverse effects of high-dose monotherapy and effectively control glycemic levels [1, 10, 11, 14]. Recently, the usage of a fixed-dose combination (FDC) has expanded due to the high compliance and cost-effectiveness of oral hypoglycemic agents [18].

There is a paucity of research on these combinations in T2DM, particularly in India. Therefore, the present study was designed to compare the efficacy and safety of triple FDC of sustained-release metformin hydrochloride 1000 mg, sitagliptin phosphate 100 mg, and pioglitazone 15 mg (MSP) with dual therapy of sustained-release metformin hydrochloride 1000 mg and sitagliptin phosphate 100 mg (MS) in T2DM patients who had failed to achieve the glycemic goal with metformin monotherapy.

## Methods

### Study design

A phase 3, randomized, double-blind, double-dummy, parallel-group, active-controlled, 24-week trial was conducted across 20 institutions in India. A total of 236 patients with T2DM were randomized between January 2022 and June 2022. The study was carried out as per the good clinical practice (GCP) guidelines and the Declaration of Helsinki ethical standards (Protocol No: ALK24-MSP1). Institutional ethical clearance (IEC) approval was obtained from all the participating sites. This trial was registered with the Clinical Trials Registry of India (CTRI/2021/10/037461).

### Study population

Patients aged 18–65 years of either gender, willing to provide informed consent and having HbA1c between 8 and 11% and with inadequate glycemic control to metformin ≥1500 mg/day for at least 6 weeks and who were capable of recording self-monitored blood glucose levels were included. Those with type 1 diabetes mellitus, FPG > 270 mg/dL, BMI ≥ 40 kg/m^2^, severe cardiovascular diseases (New York Heart Association, NYHA stages I to IV), alanine transaminase or aspartate transaminase more than three times normal, direct bilirubin ≥1.5-times normal, kidney diseases (serum creatinine ≥1.5 mg/dL), a history of malignant disease, treatment with corticosteroids or other drugs interfering with glucose metabolism were excluded.

### Intervention

After a 2-week, single-blind, run-in period, eligible patients were parallelly allocated in a 1:1 ratio by using randomization software to receive either oral FDC of 1000 mg sustained release metformin/100 mg sitagliptin/15 mg pioglitazone (Alkem Laboratories Ltd.) or an FDC of 1000 mg sustained release metformin hydrochloride/100 mg sitagliptin phosphate (brand name Janumet®, MSD Pharmaceuticals Pvt. Ltd.) once daily with breakfast. The study drugs were dispensed at each follow-up visit at 4, 8, 12, 16, 20, and 24-week intervals. Glucometer was provided to patients to self-monitor their blood glucose level twice weekly (should be at least 3 days apart) as per 5-point profile [pre-breakfast (fasting), post-breakfast (2 hours after meal), pre-lunch Post-lunch (2 hours after meal) and pre-dinner Post-dinner (2 hours after meal)], readings was recorded in patient diary to minimize and report hypoglycemia episodes if any.

### Efficacy assessments

The primary efficacy outcome was to assess the mean change in HbA1c in the FDC of MSP vs. MS therapy from baseline to 24 weeks of treatment. The secondary efficacy outcomes were to assess mean changes in fasting plasma glucose (FPG), postprandial glucose (PPG), and body weight. Moreover, we also planned to analyze the % of patients achieving an HbA1c level of < 7 in both groups post-treatment.

### Safety assessment

During the course of the trial, safety and tolerability were evaluated by monitoring vital signs, and laboratory measures included serum chemistry, hematology, and urinalysis. Twelve-lead ECG, assessment of hematology, biochemistry and urinary parameters were done at baseline, week 12 and week 24, while physical examination, vital sign measurement were carried out at all visits. Adverse events (AEs) were closely monitored and evaluated by the investigators and coded using the Medical Dictionary for Regulatory Activities (MedDRA) version 24.1.

### Statistical analysis

The minimum sample size was 236 patients (118 patients per group) with an assumed drop-out rate of 15% during the study and a power of 90% joint power for FDC of the MSP group compared with MS at the same time, 90% power for each comparison, a standard deviation of 0.8%, and a 0.4% mean difference.

Efficacy analyses were performed for all randomized patients who received at least one dose of medication. A continuous variable was represented with a mean and standard deviation (SD), and a categorical variable was represented with a frequency (%). Student’s paired t-test was performed to compare the significant difference in the mean value of both treatment groups (FDC of MSP vs. MS). Statistical analyses were performed at the 0.05 (5%) significance level. All analyses were carried out using SPSS software version 25.0 (IBM Corp., Armonk, NY, USA).

## Results

### Study population

**Fig. 1:**
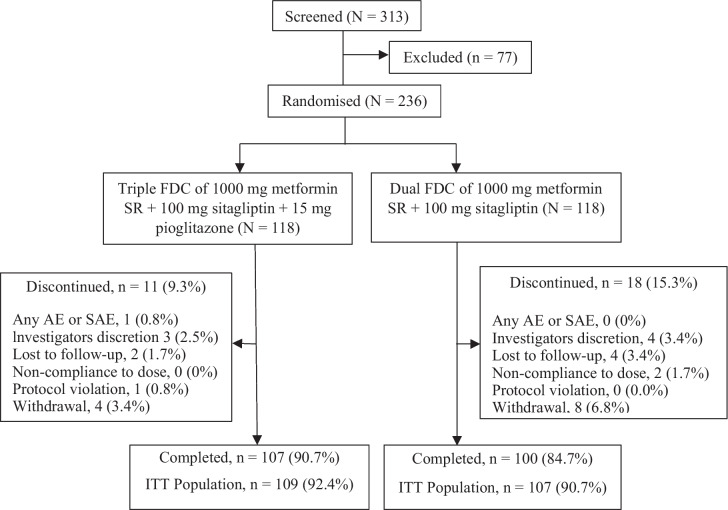
Disposition of study participants. AE, adverse events; FDC, fixed-dose combination; ITT, intention-to-treat; SAE, severe adverse effects; SR, sustained-release

The study enrolled 313 T2DM patients, of whom 236 were randomly assigned to receive either a triple FDC of MSP (*n* = 118) or MS (*n* = 118) once daily for 24 weeks. After initiating the treatment, 207 (87.71%) completed the treatment, and 29 (12.29%) discontinued it. Reasons for discontinuation were comparable between treatment groups, with a high rate of voluntary withdrawal (6.8%) observed in the MS group compared to the MSP group (Fig. 1).

The baseline and demographic characteristics were comparable between the two groups, as presented in Table 1. The study found a mean age of 51.7 years in the MSP group and 49.6 years in the MS group. The MSP group had a slightly higher BMI and a longer T2DM duration. Furthermore, the mean HbA1c was similar in both groups at randomization.

**Table 1:** Demographics and baseline characteristics of the participants

Characteristics	FDC of MSP (*N* = 118)	MS (*N* = 118)	*P* value
Male, n (%)	62 (52.5%)	63 (53.4%)	0.43
Female, n (%)	56 (47.5%)	55 (46.6%)	0.37
Age, years, mean (SD)	51.7 (8.01)	49.6 (9.54)	0.0787
Weight, kg, mean (SD)	67.1 (12.87)	65.5 (10.94)	0.3074
Height, cm, mean (SD)	162.8 (8.23)	161.5 (7.79)	0.2081
BMI, kg/m^2^, mean (SD)	25.2 (3.81)	25.1 (3.55)	0.772
Waist circumference, inches, mean (SD)	36.8 (3.62)	36.2 (3.68)	0.2106
Waist-to-hip ratio, mean (SD)	0.9 (0.07)	0.9 (0.10)	0.3733
HbA1C, %, mean (SD)	9.21 (0.78)	9.25 (0.78)	0.7181
Duration of diabetes, months, mean (SD), range	41.1 (33.47), 2–169.4	36.5 (42.86), 1.5–360.6	0.3524

FDC of MSP, fixed-dose combination of metformin, sitagliptin, and pioglitazone, MS, FDC of metformin and sitagliptin, *SD* standard deviation

### Efficacy

**Fig. 2:**
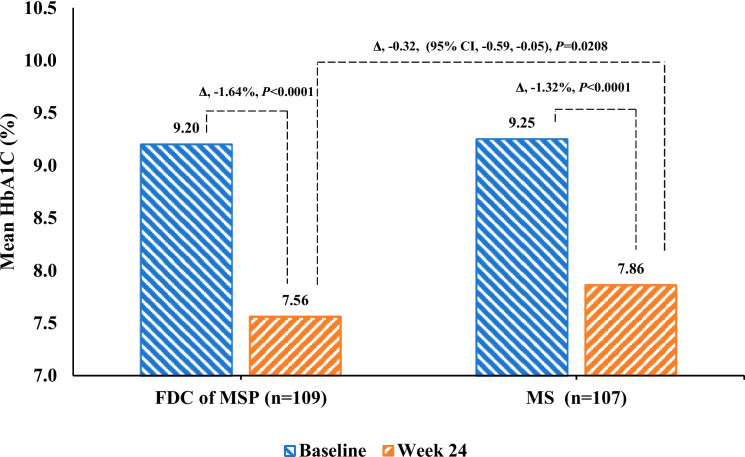
Glycosylated hemoglobin, HbA1c (%) changes from baseline to 12 weeks and 24 weeks in FDC of MSP vs. MS group

The mean change from baseline in HbA1c at week 24 was significantly greater (*P* < 0.001) in the FDC of the MSP group compared with the MS group (Table 2). In addition, a reduction in HbA1c in the FDC of the MSP group − 1.64 (95% CI, − 1.83, − 1.45) vs. the MS group − 1.32 (95% CI, − 1.52, − 1.13); least square (LS) mean difference between both groups was − 0.32% (95% CI, − 0.59, − 0.05), *P* = 0.0208 which indicates superior glycemic control in the FDC of MSP compared to the MS group (Fig. 2).

**Table 2:** Changes in glycemic parameters from baseline to 24 weeks of the trial

Characteristics	FDC of MSP (*n* = 109)	MS (*n* = 107)
**Glycosylated hemoglobin, HbA1c (%)**		
Baseline, Mean (SD)	9.21 (0.78)	9.25 (0.78)
End of the study at 24-week, Mean (SD)	7.56 (0.96)	7.86 (1.12)
LS mean change from baseline (95% CI)	−1.64 (−1.83, − 1.45)	−1.32 (− 1.52, − 1.13)
LS mean difference between the groups (95% CI)	− 0.32 (− 0.59, − 0.05)	
* P* value	0.0208*	
**Fasting plasma glucose, FPG (mg/dL)**		
Baseline, Mean (SD)	163.14 (45.05)	160.80 (44.24)
End of the study at 24-week, Mean (SD)	129.70 (30.64)	142.06 (38.76)
LS mean change from baseline (95% CI)	−31.22 (−37.88, −24.56)	−17.94 (− 24.81, − 11.07)
LS mean difference between the groups (95% CI)	− 13.28 (− 22.86, − 3.71)	
* P* value	0.0068*	
**Post prandial plasma glucose, PPG (mg/dL)**		
Baseline, Mean (SD)	234.21 (68.92)	244.85 (70.07)
End of the study at 24-week, Mean (SD)	178.75 (48.44)	198.84 (50.44)
LS mean change from baseline (95% CI)	−57.20 (−66.54, −47.86)	−36.37 (−45.81, −26.93)
LS mean difference between the groups (95% CI)	−20.83 (−34.11, − 7.55)	
* P* value	0.0023*	
**Bodyweight (kg)**		
Baseline, Mean (SD)	67.29 (12.96)	65.43 (11.08)
End of the study at 24-week, Mean (SD)	67.27 (12.52)	64.94 (10.42)
LS mean change from baseline (95% CI)	0.10 (−0.25, 0.45)	−0.34 (− 0.69, 0.02)
LS mean difference between the groups (95% CI)	0.44 (− 0.06, 0.94)	
* P* value	0.085	

*P value significance at *p* < 0.05. *CI* confidence interval, FDC of MSP fixed-dose combination of metformin, sitagliptin, and pioglitazone, LS mean, least-square mean, MS, FDC of metformin and sitagliptin, *SD* standard deviation

**Fig. 3:**
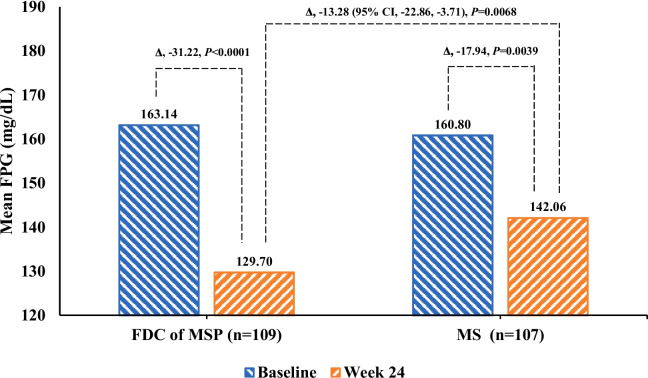
Fasting plasma glucose, FPG (mg/dL) changes from baseline to 12 weeks and 24 weeks in FDC of MSP vs. MS group

**Fig. 4:**
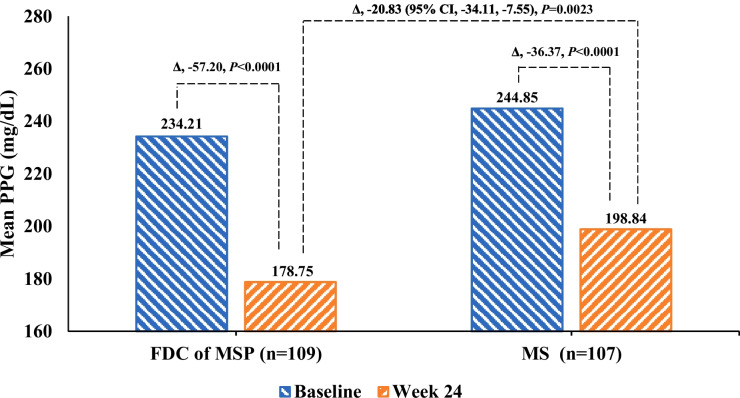
Postprandial plasma glucose and PPG (mg/dL) changes from baseline to 12 weeks and 24 weeks in FDC of MSP vs. MS group

The mean changes in FPG and 2-hours PPG were significantly (*P* < 0.001) greater in the FDC of MSP group than MS group from baseline to 24 weeks of treatment (Table 2). FPG showed a significant reduction and clinically meaningful decreases in the FDC of the MSP group vs. the MS group with a mean change of − 31.22 vs. -17.94 and LS mean difference of − 13.28 mg/dL (95% CI, − 22.86, − 3.71), *P* = 0.0068 (Fig. 3). Similarly, the study found a significant reduction with PPG difference in the FDC of MSP group − 57.20 vs. MS group − 36.37; LS mean difference between groups, − 20.83 mg/dL (95% CI, − 34.11, − 7.55), *P* = 0.0023 (Fig. 4).

**Fig. 5:**
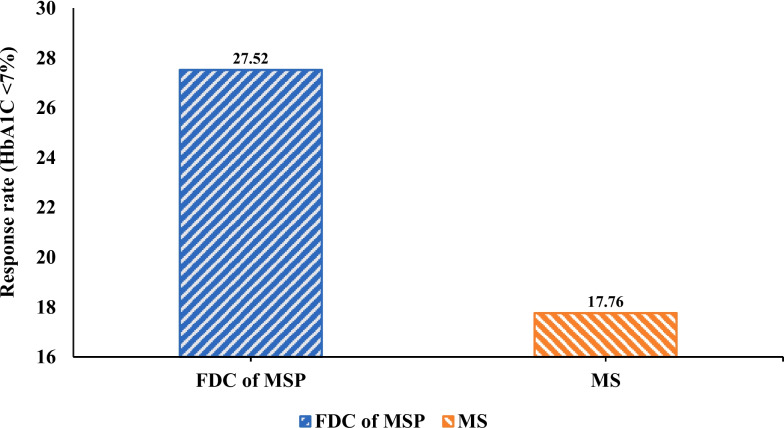
Glycosylated haemoglobin, HbA1c < 7% at 24-week in FDC of MSP vs. MS group

Figure 5 depicts the proportion of responders who achieved HbA1c levels < 7%. The FDC of the MSP group was greater 30 (27.52%) compared to MS group 19 (17.76%) from baseline to 24 weeks of treatment (*P* = 0.0866).

There was no significant LS mean body weight changes between the FDC of the MSP group and MS group from baseline to 24 weeks of treatment [0.44 kg (95% CI, − 0.06, 0.94), *P* = 0.085], as represented in Table 2.

### Safety and tolerability

Table 3 summarizes the overall adverse effects from baseline to 24 weeks of the trial. In total, 27 AEs were reported from all the participants: the incidence of AEs was numerically higher in the FDC of the MSP group 15 (12.7%) than in MS group 10 (8.5%). Most of the reported AEs were minor; one event, i.e., glomerular filtration rate 1 (0.8%), was decreased in the FDC of the MSP group, related to the investigational drug, and one severe AE, i.e., myocardial infarction 1 (0.8%), was not related to the study drug; this patient underwent angioplasty and medical management and details was recovered fully.

**Table 3:** Overview of adverse events

Characteristics	FDC of MSP (*N* = 118)		MS (*N* = 118)	
	*n* (%)	No. of events	*n* (%)	No of events
**Adverse events**	15 (12.7%)	17	10 (8.5%)	10
**Severe adverse events**	1 (0.8%)	1	0 (0.0%)	0
**Death**	0	0	0	0
**Common adverse events**				
***Blood and lymphatic system disorders***	2 (1.7%)	3	3 (2.5%)	3
Anemia	1 (0.8%)	2	3 (2.5%)	3
Thrombocytopenia	1 (0.8%)	1	0 (0.0%)	0
***Cardiac disorders***	1 (0.8%)	1	0 (0.0%)	0
Myocardial infarction	1 (0.8%)	1	0 (0.0%)	0
***Gastrointestinal***	4 (3.4%)	4	2 (1.7%)	2
Abdominal pain	0 (0.0%)	0	1 (0.8%)	1
Flatulence	2 (1.7%)	2	0 (0.0%)	0
Gastritis	1 (0.8%)	1	0 (0.0%)	0
Hyperchlorhydria	1 (0.8%)	1	0 (0.0%)	0
Vomiting	0 (0.0%)	0	1 (0.8%)	1
***Infections and infestations***	1 (0.8%)	1	1 (0.8%)	1
Nasopharyngitis	1 (0.8%)	1	1 (0.8%)	1
***Injury, poisoning and procedural complications***	1 (0.8%)	1	0 (0.0%)	0
Heat stroke	1 (0.8%)	1	0 (0.0%)	0
***Investigations***	1 (0.8%)	1	1 (0.8%)	1
Glomerular filtration rate decreased	1 (0.8%)	1	1 (0.8%)	1
***Metabolism and nutrition disorders***	2 (1.7%)	2	0 (0.0%)	0
Dehydration	1 (0.8%)	1	0 (0.0%)	0
Hyperuricemia	1 (0.8%)	1	0 (0.0%)	0
***Musculoskeletal & connective tissue disorders***	1 (0.8%)	1	0 (0.0%)	0
Myalgia	1 (0.8%)	1	0 (0.0%)	0
***Nervous system disorder***	0 (0.0%)	0	1 (0.8%)	1
Headache	0 (0.0%)	0	1 (0.8%)	1
***Respiratory, thoracic and mediastinal disorders***	2 (1.7%)	2	2 (1.7%)	2
Cough	2 (1.7%)	2	2 (1.7%)	2
***Skin and subcutaneous tissue disorders***	1 (0.8%)	1	0 (0.0%)	0
Rash	1 (0.8%)	1	0 (0.0%)	0

FDC of MSP, fixed-dose combination of metformin, sitagliptin, and pioglitazone; MS, FDC of metformin and sitagliptin

There was no change in the physical examination and vital parameters, including temperature, pulse rate, respiratory rate, systolic and diastolic BP, and clinical laboratory parameters including complete blood count, liver function test, and renal function test, in either group from baseline to all follow-up visits.

## Discussion

In the present study, the efficacy and safety of FDC of MSP (1000/100/15 mg) were evaluated over 24 weeks in comparison with a dual regimen combination of sitagliptin and metformin or MS (1000/100 mg) in patients with inadequate glycemic control on metformin monotherapy. The addition of pioglitazone to the metformin and sitagliptin regimen in FDC was well-tolerated and showed superior reductions in HbA1c, FPG, and PPG when compared to dual oral therapy.

These study results are similar to prior studies that have assessed the effects of a DPP-4 inhibitor (alogliptin) added to pioglitazone and metformin combination therapy in patients with T2DM [19, 20]. The addition of alogliptin and pioglitazone to metformin therapy was shown to result in clinically meaningful reductions in mean HbA1c from baseline (− 1.4%; *P* < 0.001). When added to metformin, the triple combination therapy of alogliptin (pooled dose; 12.5 or 25 mg) and pioglitazone (pooled dose; 15, 30, or 45 mg) was shown to be more effective than either drug in dual therapy with metformin, *P* ≤ 0.001 [20]. A recent prospective observational study conducted a specific assessment of the impact of an initial triple combination therapy, which included lobeglitazone, on drug-naive patients diagnosed. After 12 months, the study revealed that recipients of the initial triple therapy, which consisted of metformin at 1000 mg/day, sitagliptin at 100 mg/day, and lobeglitazone at 0.5 mg/day, experienced a mean reduction in HbA1c levels of 4.05% and evidenced effective efficacy and safety with the addition of lobeglitazone as part of a triple combination therapy for managing T2DM [21]. Furthermore, Bosi et al. showed an improved glycemic effect in T2DM with this triple combination therapy of gliptin, metformin, and pioglitazone, resulting in an approximately 0.7% reduction in HbA1c compared to dual regimen therapy at 52 weeks [22].

A key pathogenetic determinant underlying the deterioration of glycemic control in patients with T2DM is the progressive dysfunction of β-cell function [3]. A study revealed that pioglitazone was significantly better at alleviating insulin resistance and inferior at improving β-cell function compared with DPP-4 inhibitors in patients with T2DM under similar glycemic control [23]. Furthermore, a randomized, placebo-controlled, 26-week study was conducted with 313 T2DM patients and reported an improved glycemic effect with a good reduction in FPG and 2-h PPG levels by using triple combination therapy including sitagliptin, metformin, and pioglitazone. In addition, the study demonstrated significantly improved β-cell function, HOMA-β, and the fasting proinsulin-to-insulin ratio in triple therapy (*P* = 0.006) compared to dual therapy (*P* = 0.036) [11]. In line with these reports, the current study found effective glycemic control in the FDC of MSP therapy, which indicates the effectiveness of pioglitazone.

Despite the numerous antidiabetic medications available, weight gain remains challenging in diabetes treatment management. Obesity, especially visceral adiposity, is associated with the core defect in the pathogenesis of T2DM; the release of free fatty acids from adipocytes blocks insulin-signaling pathways that lead to insulin resistance [23]. Pioglitazone causes weight gain [11]. However, various studies reported that the addition of sitagliptin to patients already stabilized on pioglitazone did not significantly alter body weight compared with the addition of a placebo [11, 23]. Consistent with these reports, the present study does not find significant bodyweight changes with the presence of pioglitazone (FDC of MSP) compared to MS therapy.

Each drug possesses a distinct characteristic, and concurrent use of the combination of two or more drugs can affect the efficacy or could be better than the anticipated clinical outcome [24, 25]. Pioglitazone was found to be very well tolerated in a recent review of placebo-controlled, double-blind, randomized, parallel-group, multicenter clinical trials of pioglitazone administered once daily for 16–24 weeks both as monotherapy and in combination with other antihyperglycemic agents [26]. Similarly, the present study showed good tolerability with no adverse effects in either group. Moreover, the beneficial effects and favorable safety profile of triple oral therapy with pioglitazone observed in the current study are consistent with the results of a recent study in which the addition of DPP-4 inhibitors to pioglitazone and metformin led to greater decreases in HbA1c than the addition of pioglitazone alone in patients with T2DM and inadequate glycemic control on metformin [14, 17].

Despite the availability of several antidiabetic medications, patient choice is crucial when selecting medicine for chronic medical conditions such as T2DM since it involves striking a balance between effectiveness and side effects [27]. Combining two or more drug components into a single pill may enhance treatment adherence and reduce adverse effects [18, 25]. Moreover, the cost of a FDC formulation is typically similar to or lower than the combined total cost of its individual components. Limited data exist on the impact of single-pill FDCs for managing hyperglycemia on healthcare costs. A few cost-effectiveness analyses have demonstrated the clinical advantages of FDCs, showcasing reduced healthcare resource utilization and lower direct monthly healthcare expenses in clinical trials. These findings translate into cost savings and an associated increase in life expectancy [28].

Our study has some limitations. Although the study was conducted for an adequate period of 24 weeks, long-term studies could further strengthen the demonstrated results. Further assessment of the beneficial effects of triple therapy, especially pioglitazone, on insulin resistance and β-cell function would have been helpful.

## Conclusions

The study concluded that triple therapy with an FDC of metformin, sitagliptin, and pioglitazone effectively improved glycemic indices, demonstrating a good safety and tolerability profile. The FDC of MSP could be a good armamentarium for physicians to manage Indian patients with T2DM characterized by insulin resistance that is not controlled by dual therapy, as well as for those with uncontrolled diabetes who are reluctant to take insulin.

## Data Availability

The datasets during and/or analysed during the current study available from the corresponding author on reasonable request.
